# Editorial Note: Why are they eco-friendly? Attributing eco-friendly descriptive norms to intrinsic motivation increases pro-environmental purchase intention

**DOI:** 10.1371/journal.pone.0353539

**Published:** 2026-07-10

**Authors:** The PLOS One Editors

The *PLOS One* Editors issue this Editorial Note because this article [1] was identified as one of a series of submissions for which we have concerns related to the peer review process. Readers are encouraged to approach the article [1] with careful consideration.

The Editors do not have concerns regarding the authors’ conduct during the peer review process.
